# Synthesis of Silver Nanoparticles and Evaluation of Antimicrobial Activity Using the Aqueous Extract of Pterodon emarginatus Seeds

**DOI:** 10.7759/cureus.76382

**Published:** 2024-12-25

**Authors:** Maria Clara Leal Pereira, Anderson M Pereira da Silva, Léo C Magalhaes, Maria L Magalhaes, Taissa Magalhães, Eryvelton de Souza Franco, Deuzuita dos Santos Freitas Viana

**Affiliations:** 1 Department of Medicine, Center University Facid Wyden, Teresina, BRA; 2 Department of Pharmacology, Federal University of Vale do São Francisco, Petrolina, BRA; 3 Department of Medicine, Unichristus University Center, Fortaleza, BRA; 4 Department of Health Biotechnology and Pharmaceutical Sciences, Federal University of Pernambuco, Recife, BRA; 5 Department of Mechanical Engineering, State University of Piauí, Teresina, BRA

**Keywords:** antimicrobial sensitivity and resistance pattern, herbal antimicrobials, materials engineering, nano-biotechnology, nanotechnology in medicine

## Abstract

The decline in research for new antimicrobials, combined with the rise in bacterial resistance, has become a critical issue that is expected to worsen over time. As an alternative, health sciences have integrated materials engineering to develop new bioactive compounds through the interaction of nanoparticles with plant-derived compounds. These compounds offer advantages such as high bioavailability and low cost, exemplified by *Pterodon emarginatus*, a plant native to the Brazilian Cerrado. This study aimed to synthesize and stabilize silver nanoparticles (AgNPs) using the aqueous extract of *Pterodon emarginatus* seeds and evaluate their antimicrobial activity against fungal (*Candida albicans*) and bacterial (*Escherichia coli*) strains. The synthesis of AgNPs was performed using the aqueous plant extract as a stabilizing agent, with formation confirmed through UV-Vis spectroscopy, showing a characteristic absorbance peak at 400 nm. The resulting AgNPs were then tested for antimicrobial activity. While the aqueous extract of *P. emarginatus* alone showed no significant antimicrobial activity, the synthesized AgNPs demonstrated remarkable antifungal and antibacterial effects. These results highlight the synergistic interaction between the bactericidal properties of AgNPs and the bioactive compounds present in the plant extract. This approach offers a promising and sustainable alternative for the development of new antimicrobial agents, addressing the urgent need for effective solutions to combat microbial resistance.

## Introduction

The search for new antimicrobials was heavily driven by large pharmaceutical industries, particularly between the 1970s and 1990s, a period during which various drugs and drug classes were synthesized. However, in recent years, patent expirations have acted as a deterrent for these companies to invest in large-scale research aimed at developing new drugs. Combined with the growing problem of antimicrobial resistance, this scenario poses a potential risk of an antibiotic therapy collapse in the future [1,2]. 

As an alternative to this challenge, the integration of health sciences with materials engineering, particularly through nanotechnology, has emerged as a promising approach. Nanotechnology enables the manipulation of matter at the nanometric scale, allowing for atomic-level control and the synthesis of distinct structures from the same material. Within this framework, silver nanoparticles (AgNPs) have gained attention due to their remarkable antimicrobial properties and reduced toxicity compared to conventional synthesis methods [3-7]. Traditional methods for AgNP synthesis often involve multiple steps, which are not only expensive but also generate toxic and polluting byproducts. However, the use of biological extracts has been shown to provide a sustainable alternative, enabling a greener synthesis process. In addition to facilitating nanoparticle synthesis, plant-based extracts also act as stabilizing agents, enhancing their compatibility with biological systems.

This approach offers significant advantages, as plant-derived extracts are less toxic and more biocompatible, broadening their potential therapeutic applications [8,9]. Green synthesis using plant extracts leverages secondary metabolites such as flavonoids, terpenes, alkaloids, phenolics, and saccharides to reduce Ag⁺ ions to metallic silver (Ag⁰). These metabolites function not only as reducing agents but also as stabilizers for the synthesized nanoparticles. Recent studies have successfully demonstrated the synthesis and stabilization of AgNPs using biological extracts. Among the plant species investigated, *Pterodon emarginatus *(sucupira) has shown exceptional applicability [10-13].

Commonly known as white sucupira or faveiro,* P. emarginatus *(PE) is a tree native to the Brazilian Cerrado. In this context, the present study aimed to synthesize and stabilize AgNPs using the aqueous extract of *P. emarginatus* seeds and evaluate their antimicrobial activity against fungal and bacterial strains.

## Materials and methods

The aqueous extract was obtained by selecting seeds of *P. emarginatus*. Initially, 1000 mL of ultrapure water was heated in a beaker until boiling. Then, 25 g of seeds, previously weighed on a semi-analytical balance, were added to the boiling water and decocted for 10 minutes. After this period, the decoction was removed from heat and allowed to cool to room temperature. Once completely cooled, the decoction was filtered using a glass funnel and Whatman No. 1 filter paper directly into a graduated cylinder. The filtered extract was transferred to a labeled glass bottle and stored under refrigeration at 4-8 °C, protected from light. This extract was later used as a stabilizing agent in the AgNPs. AgNPs were synthesized by dripping 100 mL of a 1 mmol/L silver nitrate (AgNO₃) solution into 300 mL of a 2 mmol/L sodium borohydride (NaBH₄) solution. Subsequently, 100 mL of the aqueous extract of *P. emarginatus* was gradually added to the AgNO₃ + NaBH₄ solution. The resulting mixture was heated in a water bath at 60-70 °C, with and without stirring. The nanoparticle formation was monitored using UV-Vis spectroscopy in the wavelength range of 200-800 nm, observing a color change to yellowish-brown and a characteristic absorption peak between 400 and 500 nm.

The characterization of AgNPs was performed using UV-Vis spectroscopy with a Varian Cary 50 spectrophotometer (Agilent Technologies, Santa Clara, USA). The samples, without prior dilution or treatment, were analyzed in quartz cuvettes with a 1 cm optical path length. Absorption spectra of the solutions were recorded in the 200-800 nm wavelength range. For antimicrobial susceptibility tests, Petri plates were prepared with two layers of culture media: agar-agar and Mueller-Hinton agar. The first layer was prepared by dissolving 10.6 g of agar in 300 mL of distilled water, according to the manufacturer’s instructions. The mixture was gently stirred and heated over a Bunsen burner until completely dissolved, and then 15 mL aliquots were distributed into test tubes and sterilized in an autoclave at 120 °C for 15 minutes. After sterilization, the medium was transferred into sterile disposable Petri dishes (90 x 15 mm, Pleion®) and allowed to solidify.

The second layer, composed of Mueller-Hinton agar, was prepared by dissolving 3.6 g of the medium in 300 mL of distilled water. Aliquots of 13 mL were sterilized under the same conditions as the first layer and poured over the previously solidified plates. Once solidified, wells with a diameter of 4 mm were created using sterilized plastic tips. The strains used in the tests were* Escherichia coli *and *Candida albicans*. After microbial growth, AgNPs were applied to the wells formed in the second layer. For tests involving crude extracts, 1 mL of microbial suspension was mixed with 13 mL of Mueller-Hinton agar, maintained at 45 °C in a water bath, and poured over the base layer to solidify. Subsequently, 40 μL of the AgNP-containing solutions were carefully added to each well, following an adapted methodology described in the study by Jalal et al. [14].

The plates were incubated at 36 °C in a biochemical oxygen demand incubator for 48 hours. After the incubation period, the results were evaluated by measuring the diameter of the inhibition zones formed, using a millimeter ruler, with the values expressed in millimeters.

## Results

**Figure 1:**
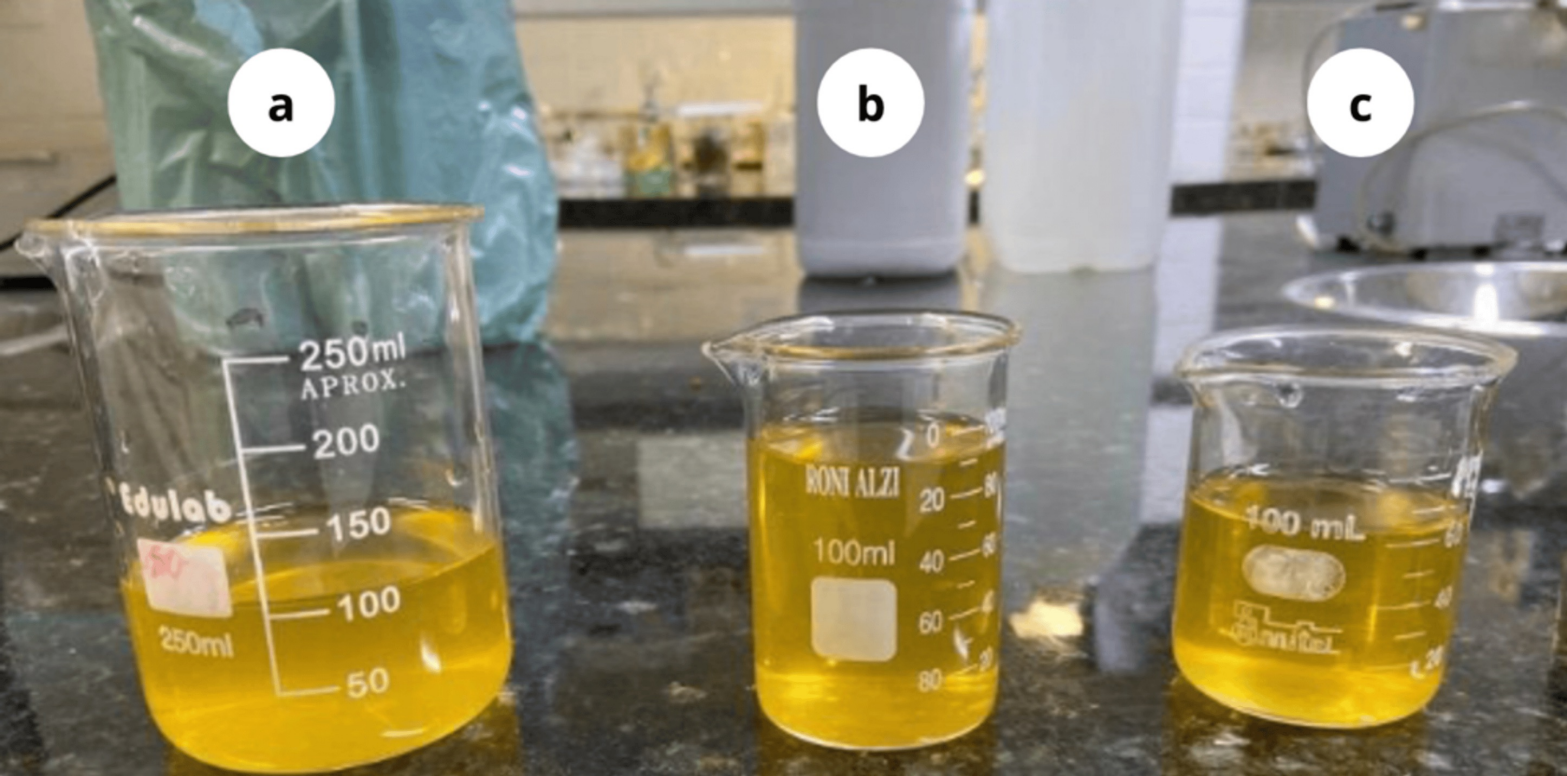
(a) 40 mL AgNPs-PE + 25 mL Scp, (b) 40 mL AgNPs-PE + 50 mL Scp, and (c) 40 mL AgNPs-PE + 75 mL Scp.

A stable solution of AgNPs with a yellowish-brown color was synthesized (Figure 1). The stability of the solution was demonstrated by the absence of nanoparticle aggregation, as they remained suspended without forming larger particles, consistent with findings in related studies [15]. During the synthesis process, NaBH₄ was added to AgNO₃, acting as both a reducing agent for silver ions (Ag⁺) and a stabilizing agent for the formed nanoparticles. 

For the experiment, 40 mL of the AgNP solution was distributed into three separate beakers. Different volumes of the aqueous sucupira extract (Scp) were added to each beaker: 25 mL (Figure 1a), 50 mL (Figure 1b), and 75 mL (Figure 1c). This procedure aimed to evaluate the effect of extract concentration on nanoparticle stabilization (Figure 1).

**Figure 2:**
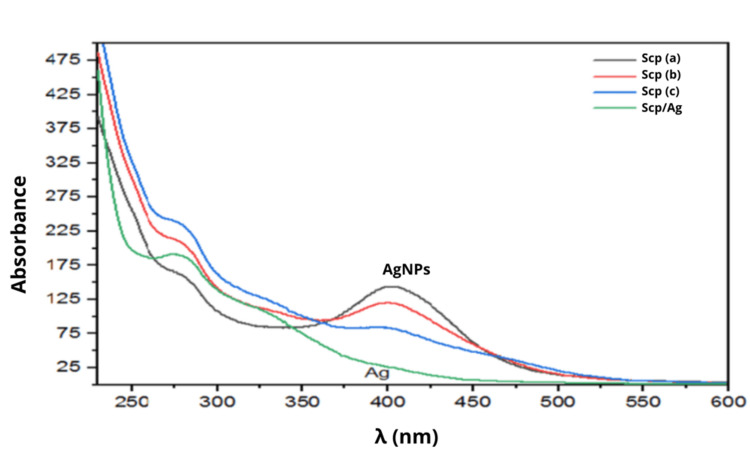
(a) 40 mL AgNPs-PE + 25 mL Scp, (b) 40 mL AgNPs-PE + 50 mL Scp, (c) 40 mL AgNPs-PE + 75 mL Scp, and (Scp/Aq) 40 mL Scp + 40 mL AgNO₃.

Subsequently, the solutions in each beaker were analyzed using UV-Vis spectroscopy. The analysis of the spectra obtained (Figure 2) revealed the formation of an absorption peak around 400 nm following the addition of NaBH₄, which is a characteristic indicator of AgNP formation, as described in the literature. However, the reaction between the aqueous sucupira extract (Scp) and AgNO₃ alone did not result in the formation of AgNPs, as no peak around 400 nm was observed [16-20].

**Figure 3:**
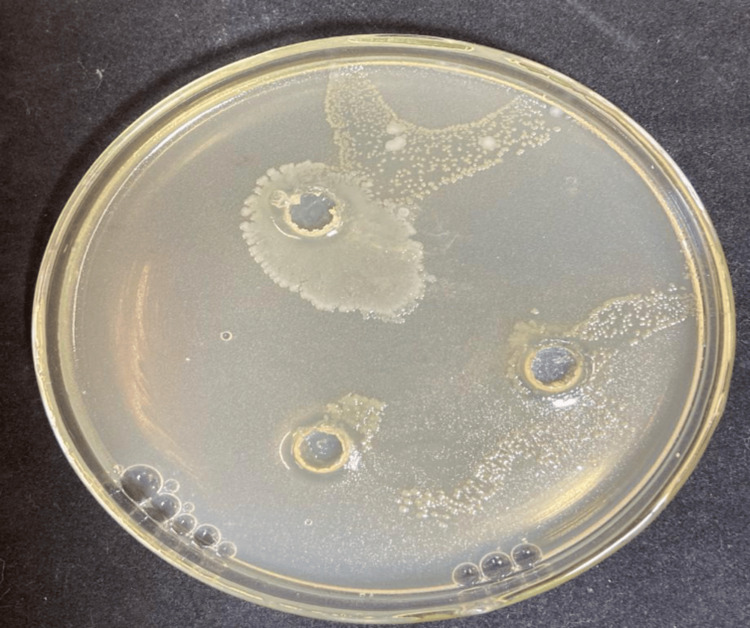
Susceptibility test of AgNPs against C. albicans.

**Figure 4:**
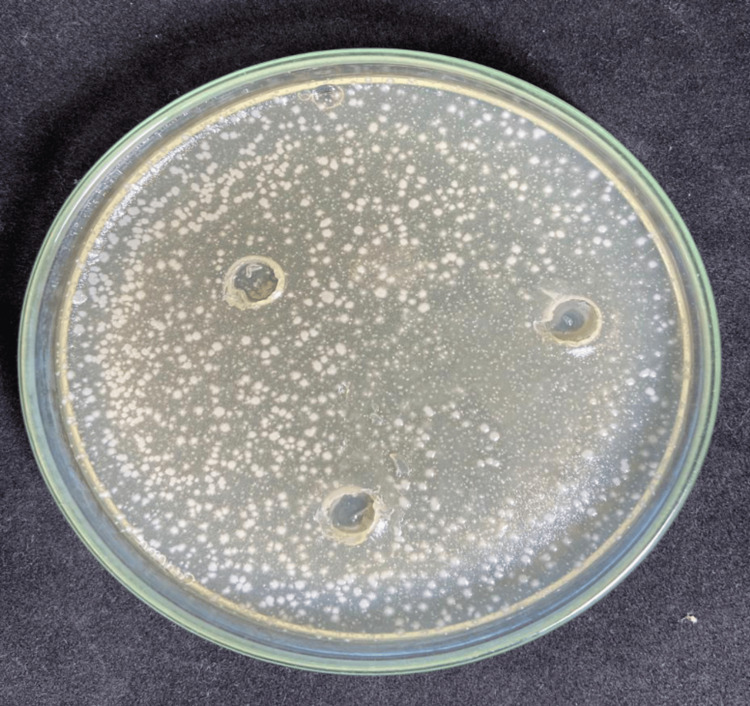
Susceptibility test of the aqueous extract of Scp against E. coli.

**Figure 5:**
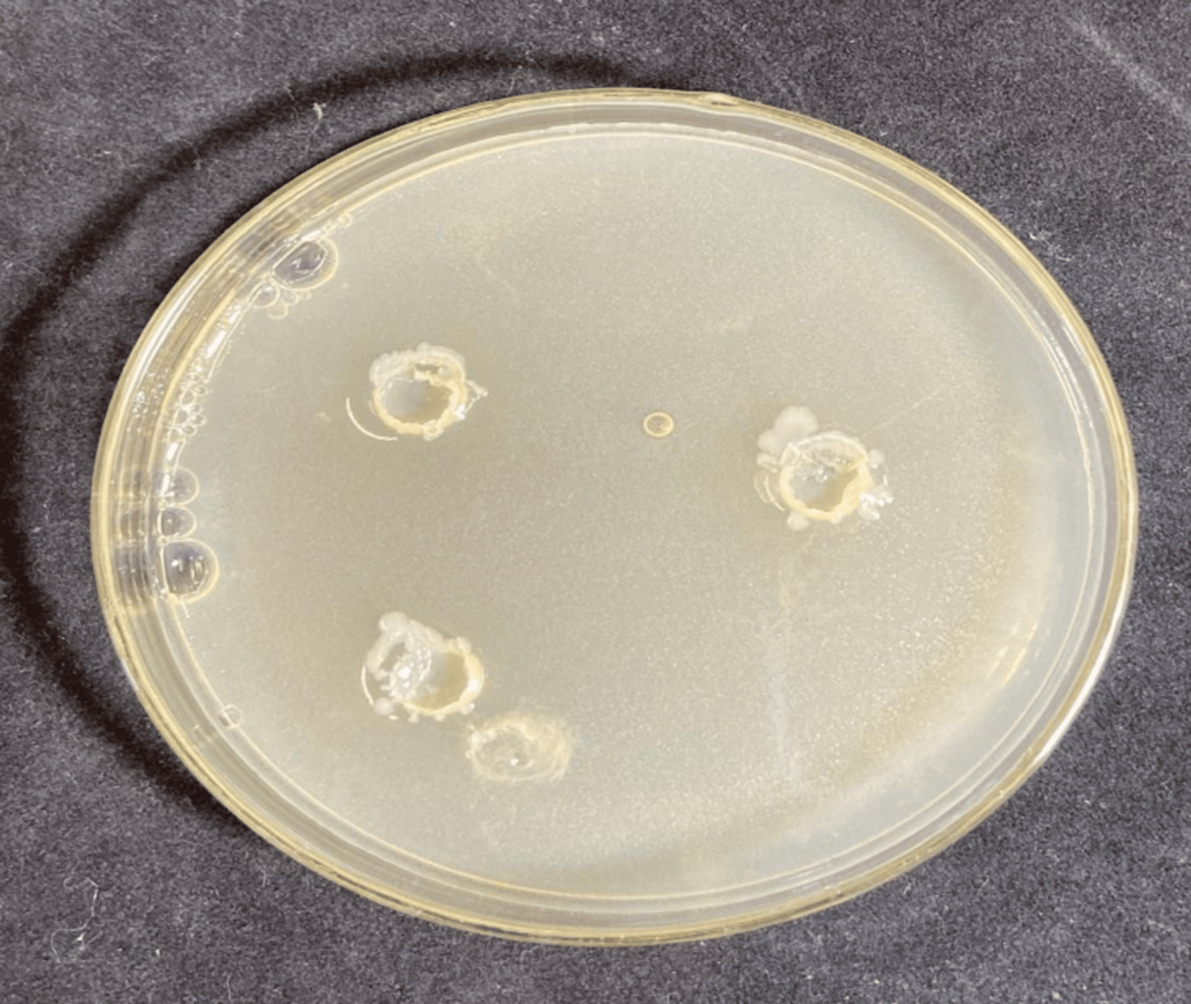
Susceptibility test of the aqueous extract of Scp against C. albicans.

**Figure 6:**
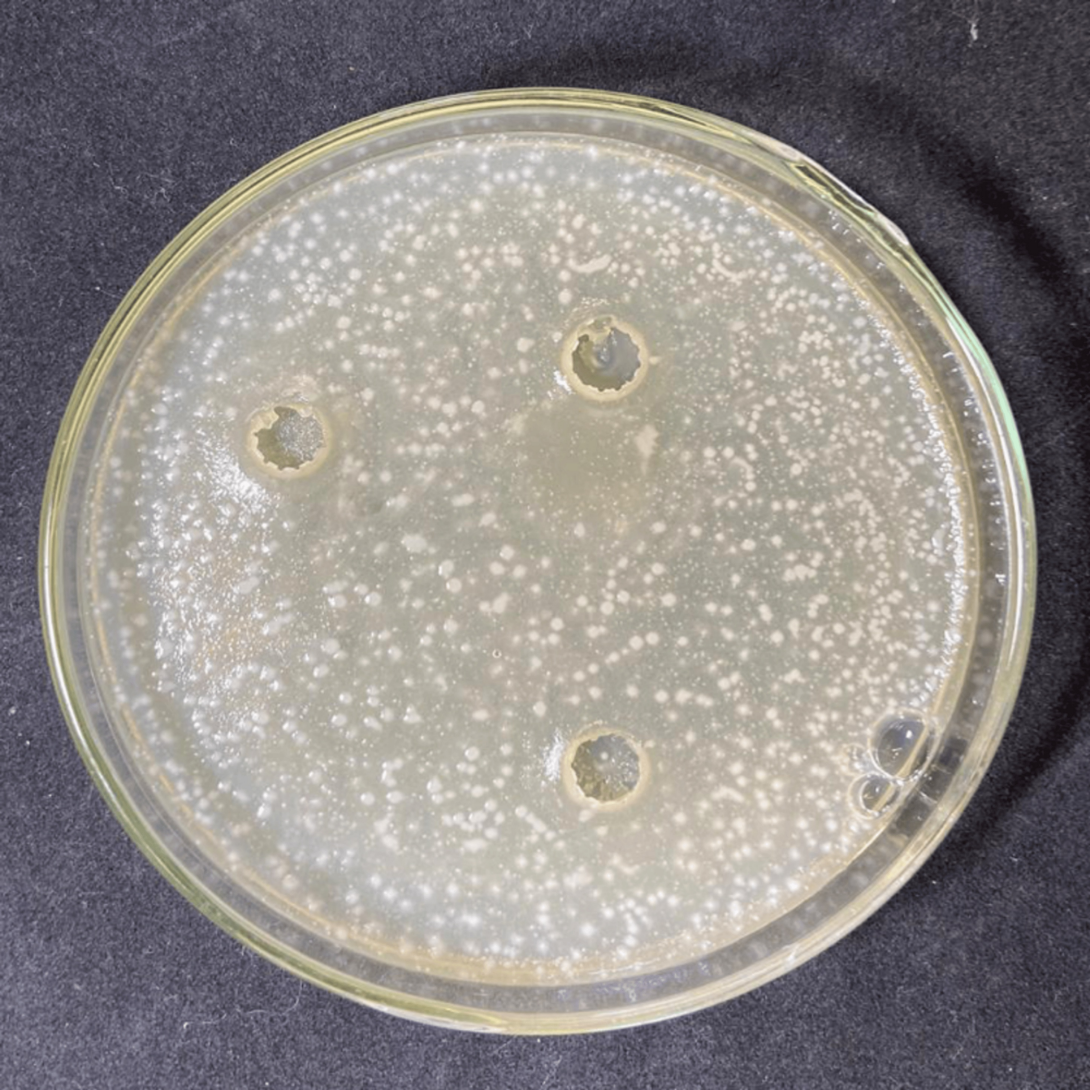
Susceptibility test of AgNPs against E. coli.

Therefore, it was concluded that the formation of AgNPs requires the addition of borohydride, and* P. emarginatus* plays a role in assisting the stabilization of AgNPs. Following this step, bacterial strains (*E. coli*) and fungal strains (*C. albicans*) were replicated, and the Scp I solution (Figure 2), which demonstrated the highest absorbance in the UV-Vis analysis, was used for the susceptibility tests (Figures 3-6).

Figure 3 highlights the susceptibility test results of the aqueous extract of Scp. The clear inhibition zones observed around the extract indicate its effectiveness in suppressing fungal growth. This result emphasizes the potential of Scp as a bioactive agent with antifungal properties.

Figure 4 demonstrates the antifungal activity of AgNPs synthesized using Pterodon emarginatus extract against C. albicans. The inhibition zones observed around the wells treated with AgNPs indicate a significant suppression of fungal growth.

Additionally, based on the analysis of Figure 5, it was inferred that the aqueous extract of *P. emarginatus* (Scp) was not effective in inhibiting the growth of the bacterial strain. In contrast, in Figure 6, the application of AgNPs resulted in the formation of bacterial growth inhibition zones with a diameter of 10 mm.

Figure 6 illustrates the antimicrobial activity of AgNPs synthesized using *P. emarginatus* extract against E. coli. The inhibition zones observed around the wells treated with AgNPs indicate a clear suppression of bacterial growth, demonstrating the effectiveness of the nanoparticles as an antimicrobial agent.

## Discussion

The study successfully evaluated the antimicrobial potential of AgNPs stabilized with the aqueous extract of* P. emarginatus* seeds. The yellowish-brown color observed in the experiment indicated the formation and stabilization of the nanoparticles, as aggregation was not observed, corroborating the findings of Rodrigues et al. [16]. UV-Vis spectroscopy analysis confirmed the formation of nanoparticles through the absorbance peaks identified in solutions with varying concentrations. It was observed that the aqueous extract of *P. emarginatus* alone was unable to induce the formation of nanoparticles, as no characteristic peak around 400 nm was detected in the absorbance analysis. Thus, the formation of nanoparticles required a reaction with sodium borohydride. However, the aqueous extract demonstrated a capacity to stabilize the nanoparticles, maintaining a stable system suitable for susceptibility testing. According to Rodrigues et al. [16], NaBH₄ reduces silver ions to metallic silver (Ag⁰) while its borohydride ions adsorb onto the nanoparticle surfaces. This adsorption generates surface charges on the nanoparticles, which create electrostatic repulsion, preventing their aggregation and ensuring the stability of the solution.

For the susceptibility tests, fungal and bacterial strains were selected. The fungal strain *C. albicans* was chosen due to its relevance in opportunistic infections, particularly in immunocompromised patients and those undergoing prolonged hospitalizations [21]. The bacterial strain Escherichia coli was selected due to its association with recurrent infections, especially urinary tract infections. In the experiments, inhibition of yeast growth was observed only in the assays using AgNPs, while the isolated application of the aqueous extract showed no antifungal activity. Similarly, for the bacterial strain, the aqueous extract alone did not demonstrate bactericidal efficacy. However, when AgNPs were applied, inhibition zones were observed. This effect is attributed to the antimicrobial properties of AgNPs, which are enhanced when associated with biological elements containing bioactive substances, such as the flavonoids and polyphenols present in the plant extract used for nanoparticle stabilization [22,23]. From this perspective, it is evident that nanoparticles combined with plant extracts containing bioactive compounds exhibit antimicrobial activity, both antifungal and bactericidal. Thus, it was inferred that AgNPs possess antimicrobial properties, which are potentiated by the synergistic interaction between the nanoparticles and the aqueous extract of *P. emarginatus*. 

The findings of this study provide a foundation for the continued exploration of AgNPs stabilized with *P. emarginatus* extract as antimicrobial agents. However, it is important to acknowledge the limitations inherent in the current experimental design. While the in vitro assays demonstrated promising antimicrobial activity, the extrapolation of these results to broader applications must be approached with caution. The use of a limited range of microbial strains and the absence of in vivo evaluations restrict the scope of the findings. To advance the potential of these nanoparticles as candidates for therapeutic or industrial applications, further studies are required, including comprehensive physicochemical characterizations, assessments of stability under diverse conditions, and in vivo testing. Standardized methodologies and expanded experimental models will be essential to confirm whether the observed antimicrobial effects can be effectively and safely translated into real-world applications. 

## Conclusions

Based on the results obtained in this study, it was possible to synthesize AgNPs stabilized with the aqueous extract of* P. emarginatus* seeds. The aqueous extract demonstrated excellent potential as a stabilizing agent for AgNPs. However, when applied in isolation during the susceptibility tests, it did not exhibit antimicrobial activity against the analyzed strains, including *C. albicans* and *E. coli.* In contrast, the AgNPs showed promising results in inhibiting both fungal and bacterial growth, clearly demonstrating their antimicrobial activity. These findings hold significant relevance for the medical and scientific community, as they may stimulate further studies on this topic. Moreover, the results provide a solid technical and scientific foundation to support the expansion of research involving nanomaterials synthesized from biological extracts.
